# Differential Effects of Extracellular Matrix Glycoproteins Fibronectin and Laminin-5 on Dental Pulp Stem Cell Phenotypes and Responsiveness

**DOI:** 10.3390/jfb14020091

**Published:** 2023-02-08

**Authors:** Hyungbin Lee, Allen Bae, John Kim, Karl Kingsley

**Affiliations:** 1 Department of Advanced Education in Orthodontics, School of Medicine, University of Nevada—Las Vegas, Las Vegas, NV 89106, USA; 2 Department of Clinical Sciences, School of Dental Medicine, University of Nevada—Las Vegas, Las Vegas, NV 89106, USA; 3 Department of Biomedical Sciences, School of Dental Medicine, University of Nevada—Las Vegas, Las Vegas, NV 89106, USA

**Keywords:** dental pulp stem cells, extracellular matrix, biomaterials, fibronectin, laminin-5

## Abstract

Dental pulp stem cells (DPSCs) are mesenchymal stem cells (MSCs) with the potential to differentiate in a limited number of other tissue types. Some evidence has suggested the modulation of DPSC growth may be mediated, in part, by exogenous extracellular matrix (ECM) glycoproteins, including fibronectin (FN) and laminin-5 (LN5). Although preliminary research suggests that some ECM glycoproteins may work as functional biomaterials to modulate DPSC growth responses, the primary goal of this project is to determine the specific effects of FN and LN5 on DPSC growth and viability. Using an existing DPSC repository, n = 16 DPSC isolates were cultured and 96-well growth assays were performed, which revealed FN, LN5 and the combination of these were sufficient to induce statistically significant changes in growth among five (n = 5) DPSC isolates. In addition, the administration of FN (either alone or in combination) was sufficient to induce the expression of alkaline phosphatase (ALP) and dentin sialophosphoprotein (DSPP), while LN5 induced the expression of ALP only, suggesting differential responsiveness among DPSCs. Moreover, these responses appeared to correlate with the expression of MSC biomarkers NANOG, Oct4 and Sox2. These results add to the growing body of evidence suggesting that functional biomaterials, such as ECM glycoproteins FN and LN5, are sufficient to induce phenotypic and differentiation-specific effects in a specific subset of DPSC isolates. More research will be needed to determine which biomarkers or additional factors are necessary and sufficient to induce the differentiation and development of DPSCs *ex vivo* and *in vitro* for biomedical applications.

## 1. Introduction

Dental pulp stem cells (DPSCs) are mesenchymal stem cells (MSCs) with the potential to differentiate in a limited number of other tissue types [1]. For example, evidence has demonstrated that DPSCs may be capable of proliferation and wound healing, as well as differentiation into osteogenic and chondrogenic lineages [2,3]. Some research has demonstrated DPSCs may have specific capabilities to facilitate differentiation into osteoblasts [4,5]. However, recent studies have suggested that DPSCs may also harbor the potential for differentiation into other lineages, separate and apart from the odontogenic and osteogenic potential for which they are well known [6].

For example, new research has demonstrated the potential of DPSCs to differentiate into bone, cartilage and fat lineages, while others have revealed the potential for neural differentiation [7,8]. In fact, evidence is accumulating that DPSCs may have vasculogenic and smooth muscle cell types [9,10]. These studies reveal the vast potential of DPSCs to mediate wound healing and facilitate tissue repair as well as their use in potential future applications in clinical therapy [11,12].

Most of the research to date has focused on inducing DPSC differentiation using a variety of exogenous growth factors [13]. For example, many studies have demonstrated the neurogenic potential of basic fibroblast growth factor (bFGF) and epidermal growth factor (EGF) alone or in combination to facilitate neurogenic differentiation [14,15,16]. In addition, platelet-derived growth factor (PDGF), transforming growth factor (TGF) and vascular endothelial growth factor (VEGF) have been shown to drive DPSC differentiation towards vasculogenesis, angiogenesis and smooth muscle cell phenotypes [17,18,19]. However, most of these efforts have focused on the potential for DPSCs towards dentinogenesis and osteogenesis [20,21,22]. In fact, many studies from this group have also explored the potential to modulate DPSC phenotypes using BMP and VEGF [23,24,25,26]. 

These studies have revealed that DPSC differentiation may be controlled not only by growth factor stimulation, but also through simultaneous interactions with the extracellular environment, including extracellular matrix (ECM) glycoproteins [27,28]. In fact, many studies now incorporate both growth factors and some type of ECM-related scaffolding to provide both types of stimulation to induce DPSCs towards specific phenotypes and differentiation [29,30,31]. This research has revealed that the ECM, alone or in combination with growth factors, may be sufficient to induce changes to DPSC differentiation and phenotypic plasticity [32,33,34]. Studies from this group have also explored the potential role of the ECM and other functional biomaterials to modulate DPSC differentiation and phenotypic plasticity [35,36]. 

Strong lines of evidence from *in vitro* cultures and the differentiation of other stem cell types have suggested this modulation may be mediated, in part, by exogenous extracellular matrix (ECM) glycoproteins, including fibronectin (FN) and laminin-5 (LN5) [37,38,39]. More specifically, laminin-5 (also known as laminin-332) has been demonstrated to induce the osteogenic and chondrogenic differentiation of MSCs *in vitro*, even in the absence of growth factor stimulation, although this line of research has not yet been replicated with DPSCs [40,41,42]. Similarly, fibronectin has also been demonstrated to modulate the *in vitro* proliferation and differentiation of MSCs, although few studies have explored these phenomena among DPSCs [43,44,45]. 

Based upon the evidence that demonstrates ECM molecules, such as laminin-5 (LN5) and fibronectin (FN), may function to modulate MSC-specific growth and differentiation responses, the primary goal of this project was to determine the specific effects of FN and LN5 on DPSC growth, viability and differentiation.

## 2. Materials and Methods

### 2.1. Human Subjects Study Approval

This study was conducted in accordance with the Declaration of Helsinki. The study procedures were reviewed and approved by the Institutional Review Board (IRB) and the Office for the Protection of Human Subjects (OPRS) at the University of Nevada, Las Vegas (UNLV) under Protocol #171612-1 “Retrospective Analysis of Dental Pulp Stem Cells (DPSC) from the UNLV School of Dental Medicine (SDM) Pediatric and Clinical Population” on 21 February 2021. 

### 2.2. Original Sample Collection Approval and Protocol

The original study protocol for the isolation and collection of DPSCs was also reviewed and approved by the UNLV IRB and OPRS under Protocol OPRS#0907-3148 “Isolation of Non-Embryonic Stem Cells from Dental Pulp” on 5 February 2010. Briefly, the inclusion criteria consisted of UNLV School of Dental Medicine (SDM) patients of record who voluntarily agreed to participate in the study and provided informed consent or pediatric assent (if under 18 years of age). Exclusion criteria included any UNLV-SDM patients who declined to participate or declined to provide either informed consent or pediatric assent.

Briefly, patients scheduled for an extraction of their premolars or third molars (wisdom teeth) as part of their orthodontic treatment were asked to participate. Following extraction, teeth were sectioned at the cementum–enamel junction (CEJ) and dental pulp was removed using an endodontic broach and placed into 1.5 mL sterile microcentrifuge tubes containing sterile 1× phosphate buffered saline (PBS) for transfer to a biomedical laboratory. DPSCs were subsequently cultured using the direct outgrowth method, as previously described [46,47]. RNA from each DPSC isolate was screened according to the guidelines by the International Society for Cellular Therapy (ISCT) for stem cell biomarkers CD73, CD90 and CD105, as well as the absence of CD45 expression [48,49]. Additional expression of stem cell biomarkers (Sox-2, Oct-4 and NANOG) was also confirmed during the initial culturing phase for a minimum of ten passages prior to cryopreservation using 10% dimethyl sulfoxide (DMSO) in media containing 80% fetal bovine serum (FBS) at 80 °C.

### 2.3. Cell Culture

Available DPSC isolates (N = 16) were thawed and placed into culture in 25 cm^2^ tissue culture-treated flasks using alpha-MEM (Minimal Essential Medium) supplemented with 10% fetal bovine serum (FBS) and 1% penicillin-streptomycin solution obtained from Fisher Scientific (Fair Lawn, NJ, USA). DPSC isolates were maintained at 37 °C in a humidified biosafety level 2 (BSL2) tissue culture chamber supplemented with 5% CO_2_. Cells were split 1:2 and doubling times were noted and compared with the original ten passages for confirmation of proliferation phenotypes, which were rapid doubling times or rDT (1–2 days), intermediate doubling times or iDT (4–6 days) and slow doubling times or sDT (10–12 days) as described in Table 1.

### 2.4. Cell Viability

Viability of the DPSC isolates was measured using the Trypan Blue exclusion assay and a TC20 automated cell counter from Fisher Scientific (Fair Lawn, NJ, USA). Briefly, Trypan Blue 0.4% from Gibco/Invitrogen (Waltham, MA, USA) was mixed with an equal volume of cell suspension and incubated for two to three minutes at room temperature in a biosafety cabinet. Cells were transferred to dual-chamber disposable TC20 cell counting slides for processing. Absolute and relative percentage of live cells were acquired, as well as cell density for both experimental and control experiments.

### 2.5. Proliferation Assays

Growth and proliferation assays were performed as previously described [35,36]. In brief, DPSC isolates were placed in 96-well tissue culture-treated plates from Corning Costar (Corning, NY, USA). Cells were seeded at concentrations of 1.2 × 10^4^ cells per well and allowed to proliferate for 24 h (1 day), 48 h (2 days) or 72 h (3 days) prior to viability assessment and subsequent fixation with 10% buffered formalin from Fisher Scientific (Fair Lawn, NJ, USA). Experimental wells were treated with 10 ng/mL of recombinant human laminin-5 (NBP256854PEP) or fibronectin (NBP261633PEP) from Novus Biologicals (Littleton, CO, USA). Following the experimental assays, viability assessment and fixation, cells were stained with Gentian Violet 1% aqueous solution from Ricca Chemicals (Arlington, TX, USA) and absorbance was read using an ELx808 BioTek (Winooski, VT, USA) plate reader at 630 nm to compare experimental and control assays.

### 2.6. Microscopy and Alizarin Red Staining

In addition, parallel experiments using six-well plates were also performed using DPSC isolates with and without the addition of laminin-5 and fibronectin. To evaluate any morphological changes and the deposition of calcium associated with osteogenic differentiation, each well was stained with Alizarin Red 1% *w*/*v* solution from ThermoFisher Scientific (Fair Lawn, NJ, USA) using the manufacturer’s recommended protocol. In brief, wells were washed with 1.0 mL of 1× PBS and then fixed. Media were aspirated and wells were stained with Alizarin Red for 15 min at room temperature. The stain was aspired and wells were washed three times with 1.0 mL of distilled water. Cells were visualized using an Axiovert inverted microscope from Zeiss (Hamburg, Germany).

### 2.7. RNA Isolation and Analysis

RNA was extracted from all DPSC isolates under experimental (laminin-5, fibronectin) and control conditions, as previously described [46,47,48,49]. Briefly, TRIzol reagent from Invitrogen (Waltham, MA, USA) was applied and the cell lysate was transferred to sterile microcentrifuge tubes with the addition of 0.2 volumes of chloroform, following the manufacturer’s recommended protocol. After incubation on ice for ten minutes, each sample was centrifuged at 10,000× *g* or relative centrifugal force (RCF) at 4 °C for 15 min. The upper RNA in aqueous phase was transferred to a sterile microcentrifuge tube with the addition of isopropanol to facilitate precipitation. Each sample was centrifuged again and the pellet washed with ethanol prior to a final centrifugation. RNA was resuspended using nuclease-free distilled water and assessed using a NanoDrop 2000 Spectrophotometer from Fisher Scientific (Fair Lawn, NJ, USA). Absorbances at A260 nm and A280 nm were used to calculate sample purity and concentration.

### 2.8. qPCR Screening

RNA from each DPSC isolate was reverse-transcribed using the Verso 1-step RT-PCR kit (AB1454LDB) from Thermo Scientific (Fair Lawn, NJ, USA) and a Mastercycler gradient thermal cycler from Eppendorf (Hamburg, Germany), following the manufacturer’s recommended protocol [48,49]. cDNA synthesis was confirmed using the NanoDrop 2000 spectrophotometer as described above. qPCR screening was performed on all samples using the SYBR Green Master Mix kit also from ThermoFisher Scientific (Fair Lawn, NJ, USA). Briefly, reactions of 20 µL were prepared using ABsolute SYBR Green Master Mix (12.5 µL), nuclease-free water (7.5 µL), forward and reverse primers (1.5 µL each), and DPSC isolate cDNA (1.0 µL) using the QuantStudio real-time PCR system from Applied Biosystems (Waltham, MA). Setting included 15 min of enzyme activation at 95 °C followed by 40 cycles of 15 s of denaturation at 95 °C, 30 s of annealing at primer-pair-specific temperature, and 30 s of final extension at 72 °C using the following validated primer sets as shown in Table 2.

The expression data from these targets were normalized to the endogenous control Glyceraldehyde 3-phosphate dehydrogenase (GAPDH) using the QuantStudio software to allow for correction of sample-to-sample variations in RT-PCR efficiency, as well as to correct for any errors in sample quantification. 

### 2.9. Statistical Analysis

Data from the RNA isolation, cDNA synthesis and proliferation assays were measured using absorbance readings. These data were compiled and summarized using Microsoft Excel (Redmond, WA, USA). Descriptive statistics including averages and ranges were compiled and comparisons between DPSC isolates, as well as between control and experimental treatments were made using two-tailed Student’s *t*-tests, which are appropriate for parametric data. Significance levels were set at alpha = 0.05.

## 3. Results

**Figure 1:**
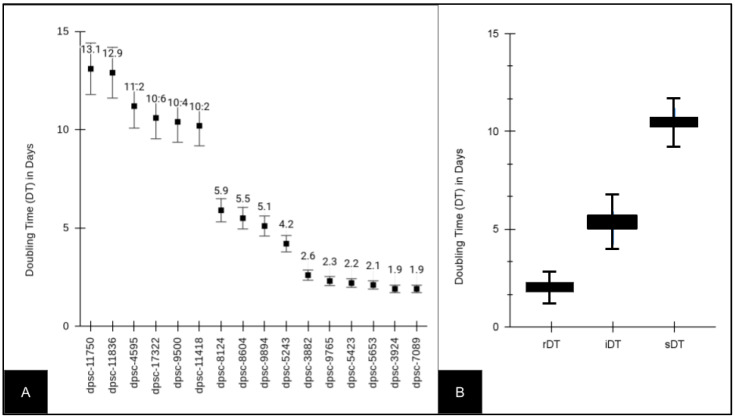
Doubling time (DT) of experimental DPSC isolates (n = 16). (**A**) Slow doubling times or sDT (10.2 to 13.1 days), intermediate doubling times or iDT (4.2 to 5.9 days) and rapid doubling times or rDT (1.9 to 2.6 days) were observed among the DPSC isolates. (**B**) rDT DPSC isolate average doubling time was 2.16 ± 0.265 days, which was different from that of the iDT DT, which was 5.18 ± 0.727 days (*p* = 0.0022), and that of the sDT DT, which was 11.4 ± 1.29 days (*p* = 0.00011).

Several DPSC isolates (n = 16) from an existing biorepository were thawed and placed into cultures (Figure 1). Several DPSC isolates (n = 6) exhibited slow doubling times (sDT) between 10.2 and 13.1 days, intermediate doubling times (iDT) between 4.2 and 5.9 days, or rapid doubling times (rDT) between 1.9 and 2.6 days (Figure 1A). A further evaluation of these data demonstrated that the rDT DPSC isolates exhibited an average doubling time of 2.16 (STD = 0.265) days, which was significantly different from the average doubling time among the iDT DPSC isolates, which was 5.18 days (STD = 0.727), *p* = 0.0022. The average doubling time of the iDT DPSC isolates was also significantly different from that of the slow DPSC isolates, which was 11.4 (STD = 1.29) days, *p* = 0.00011.

**Figure 2:**
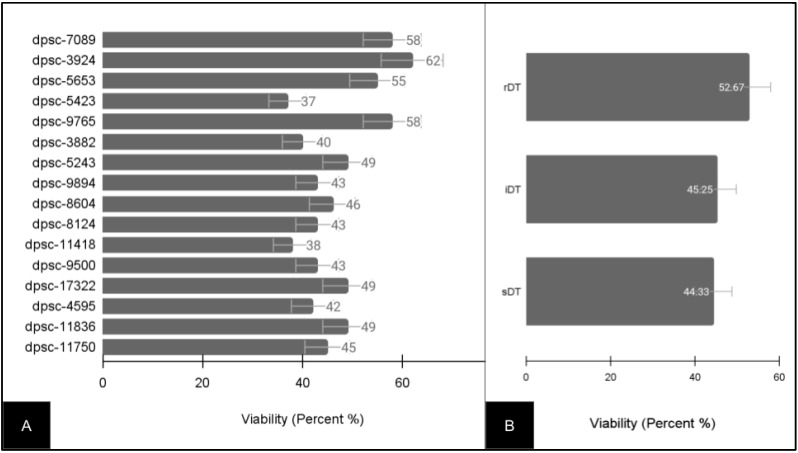
Baseline viability of experimental DPSC isolates (n = 16). (**A**) Viability ranged between 37% (dpsc-5423) and 62% (dpsc-3924), with an average of 47.31% ± 7.53. (**B**) Sorting by DPSC growth rate demonstrated rDT average viability (52.67% ± 8.41), iDT average viability (45.25% ± 7.82) and sDT average viability (44.33% ± 7.24) were not significantly different, *p* > 0.05.

Due to the variability in growth rate and doubling times, the baseline viability for all of the DPSC isolates was also evaluated (Figure 2). These data demonstrated that the viability among the DPSC isolates varied between a low of 37% (dpsc-5423) and a high of 62% (dpsc-3924) with an average of 47.31% ± 7.53 (Figure 2A). To evaluate if any differences were found between the DPSC isolates with different growth rates, the viability data were grouped and averaged by the doubling time (Figure 2B). This analysis revealed that the average viability for the rDT DPSC isolates (52.67% ± 8.41) was not significantly different from the average viability for the iDT DPSC ioslates (45.25% ± 7.82), *p* = 0.204. In addition, the average viability for the sDT DPSC isolates (44.33% ± 7.24) was not significantly different from that of the iDT (*p* = 0.695) or the rDT (*p* = 0.159) DPSC isolates.

**Figure 3:**
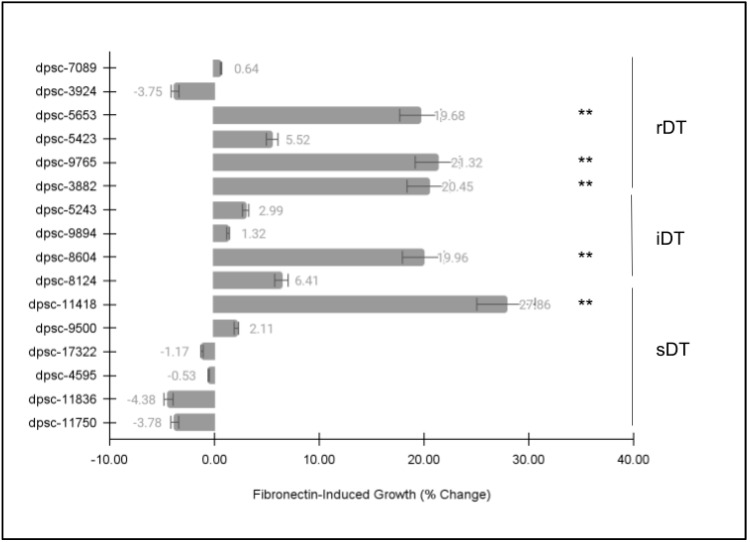
DPSC growth assay with fibronectin (FN). Five (n = 5) DPSC isolates exhibited statistically significant changes in growth under FN–induced assay conditions, including rDT DPSC isolates dpsc-5653 (+19.68%), dpsc-9765 (+21.32%), dpsc-3882 (+20.45%); iDT DPSC isolate 8604 (+19.94%); and sDT DPSC isolate: dpsc-11418 (+27.86%), *p* = 0.0000033. (** denotes statistical significance).

Each of the DPSC isolates was then plated on the extracellular matrix (ECM) glycoprotein recombinant human fibronectin (FN) to determine if there were any observable differences in cellular phenotype (Figure 3). These data clearly demonstrated statistically significant differences among some, but not all, of the DPSC isolates. More specifically, five (n = 5) DPSC isolates exhibited statistically significant changes in growth under FN-induced assay conditions. These included three rDT DPSC isolates: dpsc-5653 (+19.68%), dpsc-9765 (+21.32%), dpsc-3882 (+20.45%); one iDT DPSC isolate: 8604 (+19.94%); and one sDT DPSC isolate: dpsc-11418 (+27.86%), *p* = 0.0000033.

**Figure 4:**
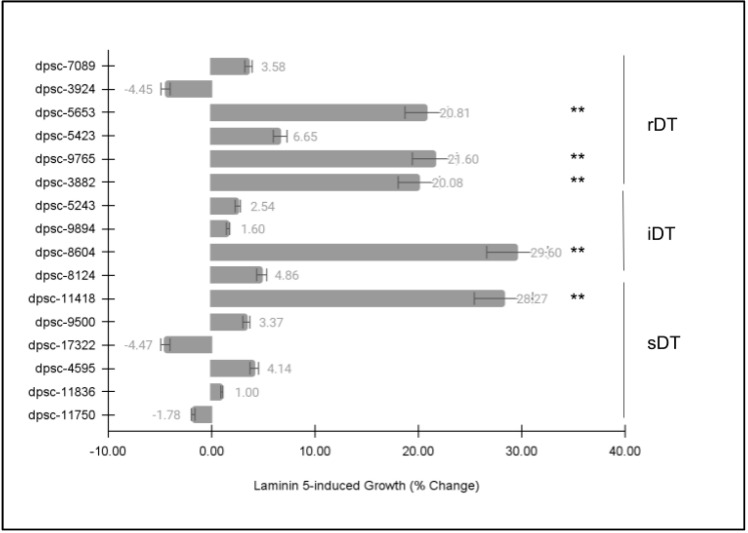
DPSC growth assay with laminin-5 (LN5). Five (n = 5) DPSC isolates exhibited statistically significant changes in growth under LN5-induced assay conditions, including rDT DPSC isolates dpsc-5653 (+20.81%), dpsc-9765 (+21.60%), dpsc-3882 (+20.08%); iDT DPSC isolate 8604 (+29.60%); and sDT DPSC isolate dpsc-11418 (+28.27%), *p* = 0.000045. (** denotes statistical significance).

Next, each of the DPSC isolates was then plated on the extracellular matrix (ECM) glycoprotein laminin-5 (LN5) to determine if there were any observable differences in cellular phenotype (Figure 4). Similar to the FN assay, these data revealed statistically significant differences among some, but not all, of the DPSC isolates. More specifically, the same five (n = 5) DPSC isolates exhibited statistically significant changes in growth under LN5-induced assay conditions. This included three rDT DPSC isolates, dpsc-5653 (+20.81%), dpsc-9765 (+21.60%), dpsc-3882 (+20.08%); one iDT DPSC isolate 8604 (+29.60%); and one sDT DPSC isolate dpsc-11418 (+28.27%), *p* = 0.000045.

**Figure 5:**
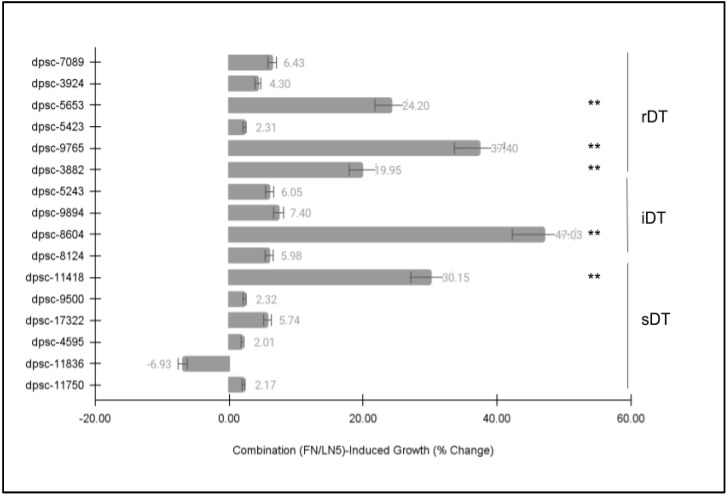
DPSC growth assay with ECM of combined fibronectin (FN) and laminin-5 (LN5). Five (n = 5) DPSC isolates exhibited statistically significant changes in growth under the combination of FN/LN5-induced assay conditions, including rDT DPSC dpsc-5653 (+24.20%), dpsc-9765 (+37.40%), dpsc-3882 (+19.95%); one iDT DPSC isolate, dpsc-8604 (+47.03%); and one sDT DPSC isolate, dpsc-11418 (+30.15%), *p* = 0.0032. (** denotes statistical significance).

Finally, each of the DPSC isolates was then plated on a combination of the extracellular matrix (ECM) glycoproteins fibronectin (FN) and laminin-5 (LN5) to determine if there were any observable differences in cellular phenotype (Figure 5). Similar to the previous ECM growth assays, these data revealed statistically significant differences among some, but not all, of the DPSC isolates. More specifically, the same five (n = 5) DPSC isolates once again exhibited statistically significant changes in growth under the ECM combination (FN and LN5)-induced assay conditions. This included three rDT DPSC isolates, dpsc-5653 (+24.20%), dpsc-9765 (+37.40%), dpsc-3882 (+19.95%); one iDT DPSC isolate, dpsc-8604 (+47.03%); and one sDT DPSC isolate, dpsc-11418 (+30.15%), *p* = 0.0032.

**Figure 6:**
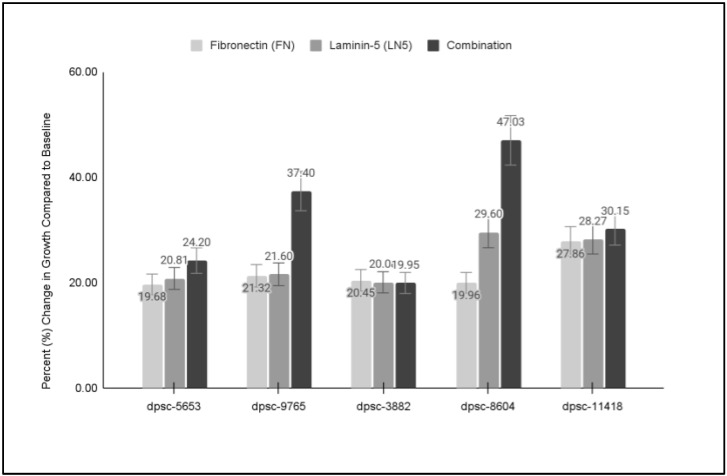
Comparison of individual and combination DPSC growth assays. Three DPSC isolates exhibited similar growth under all three experimental conditions, including rDT dpsc-5653 (19.68%, 20.81%, 24.20%), rDT dpsc-3882 (20.45%, 20.08%, 19.95%), and sDT dpsc-11418 (27.86%, 28.27%, 30.15%). However, more robust growth was observed under combination treatment with rDT dpsc-9765 (21.32%, 21.60%, 37.40%) and iDT dpsc-8604 (19.96%, 29.60%, 47.03%).

To more closely evaluate the changes in DPSC growth from these ECM-specific experiments, the proliferation data from the five DPSC isolates and the three assays (FN, LN5, Combination) were graphed and plotted for analysis (Figure 6). These data revealed that three of the DPSC isolates exhibited similar and significant increases in growth to each of the functional ECM biomaterials (FN, LN5, Combination), including two rDT DPSC isolates, dpsc-5653 (19.68%, 20.81%, 24.20%) and dpsc-3882 (20.45%, 20.08%, 19.95%), as well as one sDT DPSC isolate, dpsc-11418 (27.86%, 28.27%, 30.15%). However, two DPSC isolates exhibited a differential result with much more robust growth observed in the combination assays, including the rDT DPSC isolate dpsc-9765 (21.32%, 21.60%, 37.40%), as well as the iDT DPSC isolate dpsc-8604 (19.96%, 29.60%, 47.03%). 

**Figure 7:**
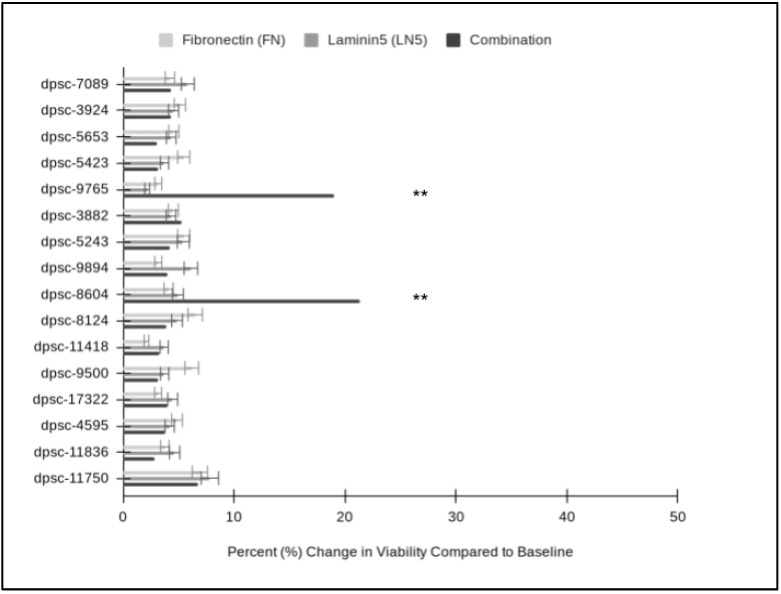
Comparison of individual and combination DPSC viability assays. FN increased cellular viability (average 4.57%; range from 2.10% to 6.19%), which was not significantly different from baseline viability or that of LN5 (average 4.67%; range from 2.16% to 7.81%), *p* = 0.832. Combination treatment increased cell viability an average of 6.01% (range from 2.82% to 6.71%) with two statistically significant exceptions: dpsc-9765′s viability increased by 18.98% (*p* = 0.033) and dpsc-8604′s viability increased by 21.30% (*p* = 0.032). (** denotes statistical significance).

To evaluate whether any of the changes in growth among the DPSC isolates in response to FN, LN5 or their combination were related to other phenotypes, the data regarding the cellular viability in all the experimental assays were compiled (Figure 7). These data clearly demonstrated that the plating of the DPSC isolates on FN had non-significant, positive effects on cellular viability which averaged 4.57% and ranged between 2.10% and 6.19%. In addition, assays with LN-5 also demonstrated positive, non-significant effects on viability, which averaged 4.67% and ranged between 2.16% and 7.81%. Finally, the combination of FN and LN5 induced positive effects on cell viability, which averaged 6.01% and ranged between 2.82% and 6.71% with two statistically significant exceptions: dpsc-9765′s viability increased by 18.98% (*p* = 0.033) and dpsc-8604′s viability increased by 21.30% (*p* = 0.032).

To evaluate if these phenotypic changes were associated with any changes in differentiation status, RNA was extracted from each DPSC isolate and converted to cDNA for a subsequent qPCR analysis (Table 3). These experiments revealed that the RNA concentrations from the DPSC isolates averaged 507.5 ± 37.93 ng/µL, which ranged between 458 and 547 ng/µL. The evaluation of the RNA purity as determined by the absorbance ratio of A260 nm and A280 nm revealed an average ratio of 1.82 with the range observed between 1.73 and 1.94. These data demonstrated that all the DPSC isolates exceeded the minimum RNA concentration and purity requirements for cDNA synthesis (as determined by the manufacturer’s protocol of 100 ng and A260:A280 > 1.65). The synthesis of cDNA revealed an average concentration of 1547.6 ± 98.3 ng/µL, which ranged between 1451 and 1641 ng/µL. The absorbance readings revealed the average A260:A280 ratio was 1.86, which ranged between 1.79 and 1.93.

**Figure 8:**
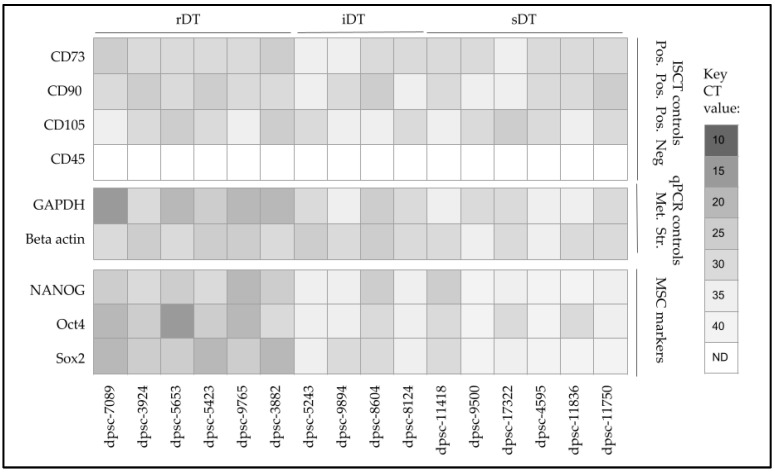
qPCR screening of DPSC mRNA for biomarkers. The expression of ISCT positive control stem cell biomarkers CD73, CD90 and CD105 was confirmed, as well as the absence of CD45. Expression of metabolic (GAPDH) and structural (beta actin) mRNA controls were observed among all DPSC isolates. The expression of MSC biomarkers NANOG, Oct4 and Sox2 was confirmed, with higher expression observed among the rDT isolates, as well as iDT dpsc-8604 and sDT dpsc-11418.

To more closely evaluate the phenotypic changes observed with these DPSC isolates, the isolated mRNA was screened using qPCR (Figure 8). These data confirmed the presence and expression of International Society for Cellular Therapy (ISCT) positive control stem cell biomarkers CD73, CD90 and CD105, as well as the absence of CD45. In addition, the qPCR results confirmed the mRNA expression of the metabolic pathway positive control Glyceraldehyde 3-phosphate dehydrogenase or GAPDH and the structural positive control beta actin among all the DPSC isolates. Finally, the expression of mesenchymal stem cell (MSC) biomarkers NANOG, Oct4 and Sox2 was confirmed, although their expression levels varied greatly. More specifically, a higher expression was observed among the rDT isolates for all three MSC biomarkers, with the differential higher expression of all three only observed among iDT dpsc-8604 and sDT dpsc-11418. Only one other iDT isolate expressed higher levels of Sox2 (dpsc-9894), while two sDT isolates expressed higher levels of Oct4 (dpsc-17322, dpsc-11836). 

**Figure 9:**
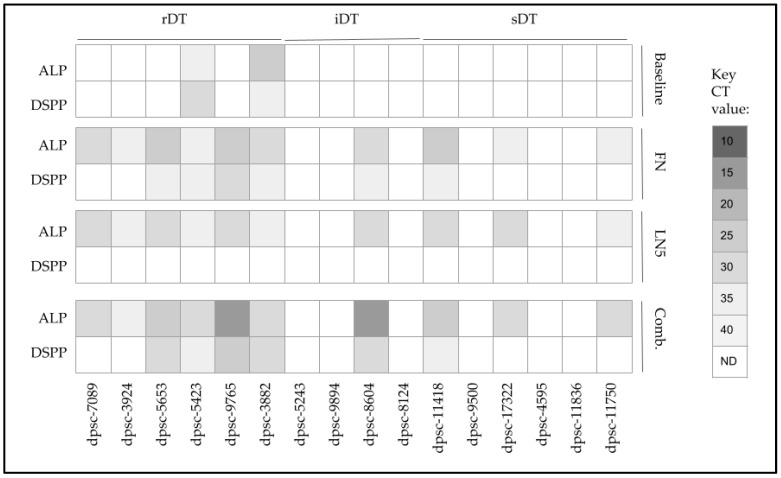
Screening of DPSC mRNA expression pre- and post-treatment. Baseline expression of alkaline phosphatase (ALP; bone and chondrocyte marker) and dentin sialophosphoprotein (DSPP, tooth biomarker) were expressed only in rDT dpsc–5423 and dpsc-3882. Administration of FN and LN5 induced expression of ALP among all rDT isolates, one iDT (dpsc-8604) and three sDT isolates (dpsc-11418, dpsc-17322, dpsc-11750). Only administration of FN or the combination of FN and LN5 induced expression of DSPP among some rDT (dpsc-5653, dpsc-5423, dpsc-9765, dpsc-3882), iDT (dpsc-8604) or sDT (dpsc-11418) isolates.

To evaluate any potential changes in differentiation towards osteogenic or chondrogenic lineages, the alkaline phosphatase (ALP) expression was evaluated along with the dentinogenesis marker dentin sialophosphoprotein (DSPP) (Figure 9). This analysis revealed that two rDT DPSC isolates already produced ALP and DSPP (dpsc-5423, dpsc-3882), although no other rDT, iDT or sDT isolates exhibited any detectable expression. However, the administration of FN was sufficient to induce the expression of ALP among all the rDT DPSC isolates, as well as one iDT isolate (dpsc-8604) and three sDT isolates (dpsc-11418, dpsc-17322, dpsc-11750). In addition, FN administration also induced DSPP expression among four rDT isolates (dpsc-5653, dpsc-5423, dpsc-9765, dpsc-3882), one iDT isolate (dpsc-8604) and one sDT isolate (dpsc-11418).

The administration of LN5 was also sufficient to induce the expression of ALP among all of the rDT DPSC isolates, as well as one iDT isolate (dpsc-8604) and three sDT isolates (dpsc-11418, dpsc-17322, dpsc-11750), similar to the effects observed with FN. However, LN5 did not induce any expression of DSPP among any DPSC isolate, while the expression of DSPP was lost under LN5 administration with dpsc-5423 and dpsc-3882. 

Finally, the combined administration of FN and LN5 was sufficient to induce the expression of ALP for all the rDT DPSC isolates, as well as one iDT isolate (dpsc-8604) and three sDT isolates (dpsc-11418, dpsc-17322, dpsc-11750), with the most robust increases observed for dpsc-9765 and dpsc-8604. In addition, the combined administration of FN and LN5 also induced DSPP expression for four rDT isolates (dpsc-5653, dpsc-5423, dpsc-9765, dpsc-3882), one iDT isolate (dpsc-8604) and one sDT isolate (dpsc-11418).

**Figure 10:**
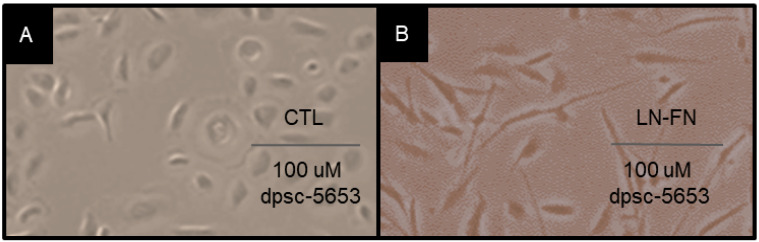
Cellular microscopy DPSC isolate dpsc-5653 under control and experimental conditions. (**A**) Untreated and control (CTL) dpsc-5653 in culture were compared with experimental ECM-treated (LN-FN) cells (**B**) demonstrating more robust proliferation under ECM treatment with some changes in cellular morphology, including extensive filopodia and lamellipodia.

To evaluate whether FN and LN5 plating was associated with any changes in cellular morphology, the cells were imaged using light microscopy (Figure 10). A qualitative analysis of the rDT DPSC isolate dpsc-5653 under the control (Figure 10A) and experimental LN-FN combination treatments (Figure 10B) demonstrated that the cellular proliferation is more robust under the ECM treatment. In addition, the DPSC under the ECM treatment exhibited more pronounced filopodia and lamellipodia, suggesting additional pathways may be activated by these experimental conditions. Additional assays confirmed the presence of calcium deposition by Alizarin Red staining (data not shown).

## 4. Discussion

The primary objective of this study was to evaluate the specific effects of the extracellular matrix (ECM) glycoproteins fibronectin (FN) and laminin-5 (LN5) on DPSC growth, viability and differentiation. This study revealed that both FN and LN5 may be sufficient to induce phenotypic changes in growth and viability among DPSCs, but these changes were not uniform among all the DPSC isolates evaluated. In addition, the evaluation of the changes in differentiation biomarkers also suggests that a subset of DPSC isolates exhibited significant proliferative responses to both FN and LN5, as well as their combination. 

These observations support the limited number of other previous studies that have found DPSCs may be growth-responsive to FN administration [50,51]. Similarly, these results also confirm observations of DPSC responsiveness to LN administration in the few studies that have been completed to date, although these studies did not specifically use LN5 [52,53]. These data add to the growing evidence that ECM glycoproteins may be a significant component of the functional biomaterials needed to induce phenotypic plasticity and differentiation among DPSCs *in vitro* [54,55].

Although many other studies have observed that the most rapidly dividing DPSCs also tend to be the most responsive to differentiation stimuli, such as the ECM, this study found that some intermediate and slowly dividing DPSCs were also capable of responding to FN, LN5 or their combination, which may be an important finding that needs to be further explored to determine the factors that govern these responses [56,57]. For example, this study found that a higher expression of the MSC biomarkers NANOG, Oct4 and Sox was observed among all of the rDT isolates, with differential higher expression of all three biomarkers only observed among the iDT isolate dpsc-8604 and the sDT isolate dpsc-11418. This correlates with the other experimental data that demonstrated the only iDT and sDT isolates responsive to FN, LN5 or their combination were dpsc-8604 and dpsc-11418. These data appear to support our previous study that demonstrated DPSC responsiveness may be related to the expression of multiple MSC pluripotency biomarkers [36]. 

This study also found that FN was sufficient to induce the expression of both ALP and DSPP among multiple DPSC isolates. This may support other lines of evidence that suggest FN may be an important mediator of dentinogenesis and tooth development [51]. However, DPSC responses to LN5 appear to only induce ALP expression, suggesting that this may be an important mediator of differentiation towards bone or chondrogenic lineages [58]. This also supports our previous work that found LN5 may be sufficient to induce osteogenic gene expression in other MSCs, although no exploration of this phenomenon has yet been conducted with DPSCs [59]. 

Although these findings have revealed significant results that demonstrate DPSC phenotypic responsiveness to ECM-specific stimuli, there are some limitations associated with the design of this study which also need to be addressed. For example, this study involved the use of an existing DPSC biorepository, which may have influenced the outcomes of the study due to the long-term effects associated with cryopreservation and storage [60,61]. In addition, this study was only able to evaluate DPSCs from a single repository; therefore, these results should be confirmed using other DPSC biorepositories and *ex vivo* samples [62]. Finally, other factors, such as the expression of microRNAs, may be important factors to consider in future studies of DPSC responsiveness to the ECM as in other recent studies of MSCs [63,64,65]. 

Finally, these results also represent part of a larger shift in research concerning DPSC differentiation and their potential therapeutic use in the field of regenerative medicine [66,67]. For example, this group has recently published preliminary data demonstrating the potential for growth factor stimulation to direct neuronal differentiation in these DPSC isolates [49]. These observations support other recent studies demonstrating significant progress towards the neuronal differentiation of DPSCs [68,69,70].

## 5. Conclusions

This study demonstrated that specific ECM glycoproteins, including FN and LN5, are sufficient to induce phenotypic plasticity among some DPSC isolates. In addition, this study found that additional factors, including MSC biomarkers, may explain, in part, these responses, although more research will be necessary to ascertain whether these factors are sufficient to induce similar responses among other populations of extracted DPSCs. These results add to the growing interest in functional biomaterials, such as FN and LN5, which are capable of modulating not only cell growth and viability but also the differentiation and gene expression of MSCs and DPSCs.

## Figures and Tables

**Table 1:** DPSC isolates.

DPSC Isolate Phenotype	Doubling Time	DPSC Isolate Reference Number
rapid doubling time (rDT) DPSC isolates	2–3 days	dpsc-7089, dpsc-3924, dpsc-5653, dpsc-5423, dpsc-9765, dpsc-3882
intermediate doubling time (iDT) DPSC isolates	5–6 days	dpsc-5243, dpsc-9894, dpsc-8604, dpsc-8124
slow doubling time (sDT) DPSC isolates	10–12 days	dpsc-11418, dpsc-9500, dpsc-17322, dpsc-4595, dpsc-11836, dpsc-11750

**Table 2:** Validated primer sequences.

Primer	Sequence
Glyceraldehyde 3-phosphate dehydrogenase GAPDH forward	5′-ATC TTC CAG GAG CGA GAT CC-3′
GAPDH reverse	5′-ACC ACT GAC ACG TTG GCA GT-3
Beta-actin forward	5′-GTG GGG TCC TGT GGT GTG-3′
Beta-actin reverse	5′-GAA GGG GAC AGG CAG TGA-3′
ISCT control CD45 forward	5′-CAT ATT TAT TTT GTC CTT CTC CCA-3′;
ISCT control CD45 reverse	5′-GAA AGT TTC CAC GAA CGG-3′
ISCT control CD73 forward	5′-AGT CCA CTG GAG AGT TCC TGC A = 3′
ISCT control CD73 reverse	5′-TGA GAG GGT CAT AAC TGG GCA C = 3′
ISCT control CD90 forward	5′-ATG AAC CTG GCC ATC AGC A-3′
ISCT control CD90 reverse	5′-GTG TGC TCA GGC ACC CC-3′
ISCT control CD105 forward	5′-CCA CTA GCC AGG TCT CGA AG-3′;
ISCT control CD105 reverse	5′-GAT GCA GGA AGA CAC TGC TG-3′
MSC biomarker Sox-2 forward	5′-ATG GGC TCT GTG GTC AAG TC-3′;
MSC biomarker Sox-2 reverse	5′-CCC TCC CAA TTC CCT TGT AT-5′;
MSC biomarker Oct-4 forward	5′-TGG AGA AGG AGA AGC TGG AGC AAA-3′
MSC biomarker Oct-4 reverse	5′-GGC AGA TGG TCG TTT GGC TGA ATA-3′
MSC biomarker NANOG forward	5′-GCT GAG ATG CCT CAC ACG GAG-3′
MSC biomarker NANOG reverse	5′-TCT GTT TCT TGA CTG GGA CCT TGT C-3′
Alkaline phosphatase (ALP) forward	5′-CAC TGC GGA CCA TTC CCA CGT CTT-3′
Alkaline phosphatase (ALP) reverse	5′-GCG CCT GGT AGT TGT TGT GAG CAT-3′
Dentin sialophosphoprotein (DSPP) forward	5′-CAA CCA TAG AGA AAG CAA ACG CG-3′
DSPP reverse	5′-TTT CTG TTG CCA CTG CTG GGA C-3′

**Table 3:** Summary of RNA and cDNA analyses.

DPSC Isolate	RNA Concentration (ng/µL)	RNA Quality A260:A80 Ratio	cDNA Concentration (ng/µL)	cDNA Purity A260:A280 Ratio
rDT isolates	506 ± 39	1.79	1586 ± 115	1.83
iDT isolates	520 ± 32	1.78	1533 ± 101	1.89
sDT isolates	500 ± 41	1.86	1524 ± 80	1.87
Average	507.5 ± 37.93 ng/µL	1.82	1547.6 ± 98.3 ng/µL	1.86
Range	458–547 ng/µL	1.73–1.94	1451–1641 ng/µL	1.79–1.93

## Data Availability

The data presented in this study are available on request from the corresponding author.
